# Building system capacity for the integration of mental health at the level of primary care in Tunisia: a study protocol in global mental health

**DOI:** 10.1186/s12913-017-1992-y

**Published:** 2017-01-17

**Authors:** Jessica Spagnolo, François Champagne, Nicole Leduc, Myra Piat, Wahid Melki, Fatma Charfi, Marc Laporta

**Affiliations:** 1 School of Public Health, Institut de recherche en santé publique de l’Université de Montréal (IRSPUM), University of Montreal, 7101 Parc Avenue, Montreal, Quebec H3N 1X9 Canada; 2 Douglas Mental Health University Institute (CIUSS Ouest-de-l’Île-de-Montréal), 6875 LaSalle boul., Montreal, Québec H4H 1R3 Canada; 3 McGill University, 845 Sherbrooke Street West Montreal, Quebec, H3A 0G4 Canada; 4 Razi Hospital, Cité des Orangers, Manouba, Tunisia; 5 Faculty of Medicine, University Tunis El-Manar, 15 rue Djebel Lakhdhar, Tunis, Tunisia; 6 Mongi-Slim Hospital, 2046 Sidi Daoud, La Marsa, Tunisia

**Keywords:** mhGAP, Mental health, Primary care, Treatment gap, Integration, Capacity-building, General practitioners, Tunisia, Effectiveness, RCT

## Abstract

**Background:**

In low- and middle-income countries (LMICs), addressing the high prevalence of mental disorders is a challenge given the limited number and unequal distribution of specialists, as well as scarce resources allocated to mental health. The Mental Health Gap Action Programme (mhGAP) and its accompanying Intervention Guide (IG), developed by the World Health Organization (WHO), aim to address this challenge by training non-specialists such as general practitioners (GPs) in mental health care. This trial aims to implement and evaluate an adapted version of the mhGAP-IG (version 1.0) offered to GPs in 2 governorates of Tunisia (i.e., Tunis and Sousse), in order to uncover important information regarding implementation process and study design before country-wide implementation and evaluation.

**Methods/Design:**

First, a systematic review will be conducted to explore types and effectiveness of mental health training programs offered to GPs around the world, with a specific focus on programs implemented and evaluated in LMICs. Second, a cluster randomized controlled trial (RCT) will be conducted to evaluate the effectiveness of the implemented training based on the mhGAP-IG (version 1.0). Third, multiple case study design will be used to explore how contextual factors impact the successful implementation of the training and desired outcomes.

**Discussion:**

In Tunisia, an important need exists to further develop proximity health services and to address the growing mental health treatment gap. One solution is to train GPs in the detection, treatment, and management of mental health problems, given their strategic role in the healthcare system. This trial thus aims to implement and evaluate an adapted version of a training based on the mhGAP-IG (version 1.0) in Tunis and Sousse before country-wide implementation and evaluation. Several contributions are envisioned: adding to the growing evidence on the mhGAP and its accompanying guide, especially in French-speaking nations; building research capacity in Tunisia and more generally in LMICs by employing rigorous designs; evaluating an adapted version of the mhGAP-IG (version 1.0) on a sample of GPs; generating important information regarding implementation process and study design before country-wide implementation; and complimenting the trial results with implementation analysis, a priority in global mental health.

## Background

Health systems around the globe are facing enormous challenges, and these are particularly apparent in LMICs [1–4]. High prevalence of mental disorders, a reliance on limited and unevenly distributed specialists, and neglect of adequate investment in resources allocated to mental health have prevented between 76-85% of people living with mental health problems in LMICs from receiving any treatment [4–9]. This treatment gap, which is on the rise in LMICs, points to the dire need of developing proximity mental health services for a population “now among the most neglected and vulnerable throughout the world” [10].

International efforts are currently invested in reforms that build system capacity in primary and community-based settings for an number of reasons [8, 11–13]. First, there are proven user and system benefits of receiving care in such settings. These include: increased user and family satisfaction with services; reduced service costs; increased access to services for a wider population; and decreased stigmatized care [9, 14–17]. Second, current reforms target primary and community-based care because improvements in mental health system capacity do not require highly specialized professionals [7, 12, 18]. Contrary to wide-spread belief on delivering mental health services, most mental health problems can be effectively managed in non-specialized health settings by non-specialists through an approach called task-sharing [2, 19–27]. Task-sharing is defined as “moving the primary provision of the mental health intervention from mental health specialists (e.g., psychiatrists, psychologists, Master level providers) to lay counselors (i.e., limited to no mental health training or experience)” [25]. International efforts are assuming this approach because of its concordance with the realities of LMICs - it emphasizes the need to involve primary healthcare professionals and/or the lay workforce given the limited number and unequal distribution of mental health specialists [5, 18, 24, 25, 28].

GPs have been targets of many task-sharing initiatives worldwide because they are ideally placed in the healthcare system [29–31]. However, they often lack appropriate knowledge and skills to adequately detect, treat and manage mental health problems. To respond to this gap in knowledge, a number of mental health training programs targeting GPs have been developed and implemented worldwide. Such trainings contribute to health system reform in that “there is evidence that adequate training can reduce variations in provider behavior, improve fidelity, and ultimately increase the quality of service delivery” [32]. Developing and implementing mental health trainings that seek to build capacity and further integrate mental health into routine general practice has also been identified as a priority in global mental health [33].

It is important to note that questions regarding evidence on building mental health system capacity by offering training programs to non-specialized healthcare professionals, including GPs, often arise. First, findings are mainly from high-income countries (HICs) [7, 26, 34] and do not concord with the realities of LMICs due to differing culture and context, preventing the uptake of relevant and useful knowledge in these settings [34]. Therefore, generating appropriate and usable knowledge is an increasingly important research priority in global mental health [7, 26, 34, 35]. Second, most mental health training programs are focused solely ﻿on﻿ evaluating effectiveness or efficacy using experimental trials such as RCTs, which are known to disregard contextual factors that might influence the uptake and use of knowledge, practice-level changes, system-level changes, and sustainability of an implemented program [34, 36]. Therefore, implementation analysis is needed because﻿ it highlights how culture and context affect the successful implementation of an intervention within a dynamic environment, which can have a significant impact on desired training outcomes [36]. Last, most mental health training programs are not designed in the form of a ‘package,’ where training is complimented with guidelines that seek to develop mental health policies and systems [17, 26]. These guidelines are important because they can help decision-makers orchestrate and sustain reforms [7, 26, 37].

In 2008, the WHO launched the mhGAP in response to these gaps in evidence on building mental health system capacity. The Programme aims to train non-specialists in mental health detection, treatment, and management, all the while complimenting training with discussions around implementation, as well as system and policy development [26, 38]. In 2010, the mhGAP Intervention Guide (IG), currently in its second edition, was developed to encourage delivery of evidence-based interventions for what the WHO deems priority mental disorders [2, 39, 40]. The guide was developed by systematically searching the literature on ways to effectively treat and manage mental disorders in non-specialized settings by non-specialists [2]. Interventions included in the guide were also subject to international expert consultation [2].

The mhGAP-IG is the current mental health training of choice around the world for a number of reasons. Unlike previous mental health trainings, the evidence is based on findings specifically from LMICs, as well as expert opinion from researchers, decision-makers, and healthcare professionals working within these countries [26, 34, 39]. In addition, the mhGAP-IG was developed through international participatory consensus-based processes [39]. Participatory processes are particularly important when developing training interventions for mental health seeing as “the classification system for mental disorders that will be satisfactory for primary care must capture the complexity of the range of presentations of psychological problems in that setting” [39]. For the above mentioned reasons, the mhGAP-IG was chosen as the intervention for this trial.

The Tunisian Ministry of Health, in collaboration with the School of Public Health at the University of Montreal, the WHO office Tunisia, and the Montreal WHO-PAHO Collaborating Center for Research and Training in Mental Health (Douglas Mental Health University Institute), is interested in implementing an adapted version the mhGAP-IG in 2 governorates (i.e., Tunis and Sousse), in response to a country-wide health services reform that began in 2013. One of the main targets of this reform is to strengthen health system capacity by creating proximity health services [41, 42]. This reorganization aims to: 1) promote the use of multidisciplinary teams in primary care settings; 2) valorize general medical practice; and 3) equip primary care practitioners in effective patient management [42]. This reform is also discussed extensively to meet the needs of people living with mental health problems in Tunisia [41].

Implementing a mental health training based on the mhGAP-IG (version 1.0) thus comes at an opportune time during the health systems reform in Tunisia. Although Tunisia is equipped with mental health services, they are mainly provided in the capital (through the only standing and overcrowded mental health hospital in the country) and along the coastline (through psychiatric units within regional hospitals), making the distribution of resources uneven and impeding on equal access to services [43, 44]. In addition, Tunisia suffers from a shortage of mental health professionals, such as psychiatrists, psychologists, psychiatric nurses, and mental health social workers [41, 43] also echoed in many other LMICs. Shortages of mental health specialists in Tunisia force non-specialists such as GPs to receive between 30–40% of mental health consultations, despite their limited ability to adequately detect, treat, and manage mental health problems in primary care [41, 45].

## Objectives

This trial aims to implement and evaluate an adapted version of the mhGAP-IG (version 1.0) offered to GPs in 2 governorates of Tunisia (i.e., Tunis and Sousse), in order to uncover important information regarding implementation process and study design, before country-wide implementation and evaluation. The main objective of the trial is divided into 3 phases:

Phase 1 aims to answer the following research question by conducting a systematic review: 1) What types of mental health training programs offered to GPs have been implemented and evaluated, and are they effective? This review, which to our knowledge has not yet been previously conducted, will: 1) help us﻿﻿ gain a broader perspective on tested training outcomes, in order to inform this trial; 2) compliment already available findings on the mhGAP-IG; and 3) compare the effectiveness of a mental health training based on the mhGAP-IG (this trial) with previously implementing training programs in LMICs.

Phase 2 aims to answer the following research question by conducting a cluster RCT: 2) What is the potential value of building capacity in primary or community-based settings by training GPs in Tunis and Sousse (Tunisia) using the mhGAP-IG? Five specific modules from the mhGAP-IG (version 1.0) have been chosen by members of the Ministry of Health in Tunisia to reflect current and pressing needs: depression; psychosis; suicide/self-harm; alcohol use disorders; and drug use disorders. The main hypothesis of this cluster RCT is that the mental health training based on the mhGAP-IG will be clinically useful; will improve/increase GPs’ knowledge about disorders selected for training, attitudes towards mental illness, and perceived clinical self-efficacy; and will improve/increase rates of detection, treatment and management of mental illness. In addition, the cluster RCT will allow us to obtain crucial information on the design, namely the acceptability of delivering the mental health training as planned for the trial, as well as the estimated effect size and intra-cluster correlation (ICC) of a mental health training based on the mhGAP-IG. At the time this protocol was written and defended (June 2015), this information was not available.

Phase 3 aims to answer the following research question by multiple case study design: 3) How do contextual factors influence the successful implementation and expected outcomes of a mental health training based on the mhGAP-IG (version 1.0) offered to GPs in Tunis and Sousse (Tunisia)? This type of evaluation is referred to as Type III implementation analysis [36] and is currently a priority in global mental health [34].

## Methods/Design

### Phase 1: Conducting a Systematic Review

#### Search strategy and data collection

A systematic review will be conducted to explore the types and effectiveness of mental health training programs offered to GPs worldwide, with a specific focus on primary care in LMICs. To our knowledge, this is the first systematic review on the topic, and will be used to improve the training intervention offered to GPs in Tunis and Sousse. It will also compliment findings on the mhGAP-IG.

JS met with a librarian at University of Montreal to generate a search strategy for this review, which is currently underway. To answer the research question, the following databases are currently being searched: MEDLINE, PubMed, Embase, CINAHL, PsycINFO, and Web of Science. The main search terms used to generate the search strategy include: general practitioners; primary care; mental health; mental disorders; psychiatry; training programs; and education. Google will be used as a means to find grey literature. Once articles have been selected, reference lists will be searched for additional eligible articles. After indentifying the articles to be included in this review, key individuals in the field of capacity building by training GPs in mental health detection, treatment, and management will be contacted to validate findings and/or to obtain information on additional publications.

#### Study selection

Study eligibility criteria has been developed. These include: 1) academic and grey literature published from 1978 onwards; 2) articles written in English, French, and Spanish; and 3) study designs including RCTs, cluster RCTs, and quasi-experimental designs, to match our trial design. Studies will be excluded if they do not have a control/comparison group, and if they are descriptive or qualitative only.

#### Data analysis

Titles and abstracts of articles found using the search strategy will be reviewed. If they meet eligibility criteria, full texts will be obtained. Full texts will be included only if they meet eligibility criteria after review. Included texts will be reviewed for quality to deem if the training programs are effective.

Quality will be assessed using the *Quality Assessment Tool for Quantitative Studies* (1998) (http://www.ephpp.ca/tools.html) [46]. It was developed by *Effective Public Health Practice Project* (EPHPP) and specifically designed for use in public health. According to Jackson & Waters (2005) [47], this tool is considered adequate for analyzing articles that target interventions. Six content areas are included: allocation bias; confounders; blinding; data collection; as well as withdrawal and dropouts. Each of the content areas are rated as such: strong (3 points), moderate (2 points), and weak (1 point), for a maximum of 18 points per study analyzed. Content area scores are then averaged to provide the overall quality score [48].

Studies show that this quality tool has acceptable internal consistency and test-retest properties [47]. The *Quality Assessment Tool for Quantitative Studies* [46] is accompanied by a reviewer’s dictionary to ensure standardized use.

### Phase 2: Building mental health capacity by training GPs in Tunisia

The method section below follows the SPIRIT Guidelines.

### Participants, interventions and outcomes

#### Study setting

To assess the potential value of capacity building by training GPs in Tunis and Sousse using an adaptation of the mhGAP-IG (version 1.0), a cluster randomized controlled trial (RCT) with two arms (i.e., intervention and control) will be conducted. Tunis and Sousse have been chosen as they regroup the majority of the Tunisian population; they have access to the only standing mental health hospital in the country, as well as psychiatric units located in general hospitals; and in this area, there are substantially more resources allocated to mental healthcare (i.e., doctors, clinics, medication) than in other areas of Tunisia. Delegations (i.e., designated areas within the governorates) have been chosen as the clusters for this trial, seeing as health services are organized accordingly in Tunisia. There are 22 delegations in Tunis and 16 in Sousse, for a total of 38 delegations.

#### Eligibility criteria

The group of participants who will be recruited for this trial are GPs working within private or public institutions at the level of primary care in Tunis or Sousse. GPs will be recruited by identified clinicians working to promote continuing medical education in Tunis and Sousse. These clinicians, who work within private or public institutions at the level of primary care, have been selected by members of the Ministry of Health in Tunisia to be a part of this trial, as they have advanced knowledge and skills in the field of mental health, and they are mandated to encourage continuing medical education within their respective delegations. GPs will also be approached by 1 psychiatrist-trainer, as she works closely with GPs within the community.

To be included in the trial, GPs must meet the following eligibility criteria: 1) working within public or private institutions at the level of primary care in Tunis or Sousse; 2) having 5 or more years of clinical experience; 3) dedicating a minimum of 1 h per week to mental health; 4) being part of the *Conseil national de l’ordre des médecins de Tunisie* (CNOM), which is the GP order in Tunisia; and 5) being available when the training is scheduled. GPs will be excluded from the trial if they are retired or on sick leave; work in any other setting than in primary or community-based institutions; or do not dedicate any time to mental health or illness within their given work-week.

#### Interventions

The training intervention is based on an adapted version of the mhGAP-IG (version 1.0) developed by the WHO [2]. Instead of implementing all the suggested modules of the mhGAP-IG (version 1.0), 5 modules have been chosen for the purposes of this trial by members of the Ministry of Health in Tunisia: depression; psychosis; suicide/self-harm; alcohol use disorders; and drug use disorders. In addition to these modules, general principals of care as well as an introduction to the mhGAP will be presented.

Using the mhGAP Adaptation Guide developed by the WHO, the training modules and the accompanying training material (PowerPoint, trainer and participant guides) will be adapted to the local primary care context of the 2 governorates.

The training will be conducted by 3 Tunisian psychiatrists, trained in the proper use of the mhGAP-IG. The mhGAP training for participating GPs will take place one afternoon a week, over 5 weeks. A total of 17.5 h (3.5 h a week) is envisioned for the training modules, followed by a 2-h supervision session. During the supervision session, participating GPs will be invited to present mental health cases to the trainer-psychiatrists, engage in additional role plays, and review some of the material presented during the training sessions.

To improve adherence, participating GPs will be given an attestation signed by the President of the Committee for Mental Health Promotion in Tunisia, certifying that they have completed the training program.

#### Outcomes

Outcomes include GPs’ knowledge about disorders selected for training, attitudes towards mental illness, and perceived clinical self-efficacy in detecting, treating, and managing patients with the selected disorders.

#### Sample size

This trial will answer a number of important questions regarding study design, namely: What is the estimated effect size and ICC of a mental health training based on the mhGAP-IG? These parameters, to our knowledge, were not available at the time this protocol was written and defended (June 2015), and will thus make significant contribution to knowledge on the mhGAP-IG.

Following consultation with members of the Ministry of Health in Tunisia, the recommended average number of GPs to be recruited in the cluster (i.e., the delegation, many of which comprise the governorate) was suggested to be 15. While some studies using a cluster RCT to evaluate the effectiveness of a mental health training program offered to GPs in HICs do not report attrition [49–51], we are concerned that the evidence does not reflect the sampling realities in LMICs. For this reason, we aim to recruit 19 GPs per delegation, to be sure we account for a maximum of 20% attrition rate per cluster [52, 53]. Table 1 highlights the estimated sample size and number of clusters for the trial.

**Table 1:** The sample size and number of clusters in the trial

PARAMETERS	#
n (total number of GPs)	722
# clusters (delegation)	38
n cluster (GPs on average per cluster)	19

Using the statistical software G*Power 3.1, the effect size can be calculated after data collection. Parameters will be set at: 1) test family: *t* test; 2) statistical test: difference between two independent means; 3) tail(s): two-tailed test; 4) type of power analysis: sensitivity; 5) alpha: 0.05; 6) power: 0.80; and 7) sample size (i.e., the total number of GPs) for control and intervention groups used in this trial. Once the effect size is found, the estimated ICC can be generated using the following formula, designed for cluster RCTs: *N =* N_sg_ (1 + (m-1) ICC), where:

- *N =* number of participants in the trial (i.e., the total number of GPs);
- N_sg_ = number of participants in the trial, without considering clusters;
- m = number of participants in the cluster (i.e., the average number of GPs in the cluster);
- ICC = intra-cluster correlation (i.e., the correlation among GPs in the cluster).

#### Recruitment

GPs will be recruited by identified clinicians working to promote continuing medical education in Tunis and Sousse. A training on the description of the study and participant requirements will be given to the identified clinicians before recruitment phase. Identified clinicians will then collect the names and contact information of the interested participants, who will be contacted by JS to obtain written consent before randomization.

### Assignment of interventions

#### Allocation sequence generation

A randomization scheme must be generated to randomize the delegations either to the intervention or control group. Using SAS software version 9.3, a random seed (*blockrand* function) will be used to produce simple randomization by fixed blocks of 3. A list of these simple blocks will be used to determine the delegation assignment.

#### Allocation concealment mechanism

All GPs working in the delegations included in this trial will be offered the training, but at varying times. Therefore, it will be impossible to determine which delegation (and thus participating GPs) is assigned to either the intervention or control groups. Psychiatrist-trainers, clinicians responsible for GP recruitment, members of the Ministry of Health in Tunisia, and directors of the delegations included in this trial will not be informed of the allocation.

#### Implementation

JS will be responsible for the overall management of the trial, including the generation of the allocation sequence, and assignation of delegations to either the intervention or control group. While in Tunisia, JS will be working under the auspices of members of the Ministry of Health and the WHO office. They will help ensure the successful implementation of the training program in Tunis and Sousse.

#### Blinding

To protect against result contamination, delegations and not individuals will be randomized. Given the geographic distance between each delegation included in this trial, it is very unlikely that GPs from different delegations will share information during and after the training sessions. Selection bias will be avoided by randomization.

Members of the Ministry of Health and WHO office in Tunisia working to ensure the successful implementation of this training program in Tunis and Sousse will be blinded to the allocation of delegations.

### Data collection, management, and analysis

#### Data collection and methods

Questionnaires will be administered to the intervention and control groups at different times. These include questionnaires on socio-demographics, knowledge, attitudes, self-efficacy and mental health practice. The socio-demographic questionnaire will include information on GPs’ sex, age, number of years working in primary care, percentage of time dedicated to mental health in primary care, education, previous mental health training, and work location.

The knowledge questionnaire has been developed by the WHO to accompany the mhGAP-IG and training package. However, it has been adapted to conform to the modules that have been chosen for the purposes of this trial.

The *Mental Illness Clinicians’ Attitudes (MICA) Scale (version 4)* [54, 55] was chosen to assess GPs’ attitudes in this trial. This scale is a modified version of the *Mental Illness Clinicians’ Attitudes (MICA) Scale (version 2)*, which aims to assess attitudes of medical students towards mental illness and the mental health field. Kassam et al. (2010) [54], by modifying this scale, developed a version that can be used with students and health care professionals of any health discipline. It is of interest for this trial because most of the other scales that aim to assess health professionals’ attitudes towards mental illness have questionable psychometric properties [55]. The *MICA Scale (version 4)* has 16-items, and answers range from ‘strongly agree’ to ‘strongly disagree’. It was shown to have adequate internal consistency and test-retest properties [55].

A self-efficacy questionnaire was developed for the purposes of this trial. Self-efficacy is a concept first introduced by Bandura [56], and is part of his social cognitive theory as a “key psychological construct with regards to how people adapt to their environments where new skills are developed” [57]. More specifically, self-efficacy refers to people’s beliefs in their capabilities, which influence performance attainment, achievement of outcomes, and behavioural change [56–58]. For these reasons, assessment of perceived clinical self-efficacy is of interest when evaluating training programs because positive effects on self-efficacy scales should translate into practice change [59]. Bandura (2006) [58] suggests that the best way to measure self-efficacy in a study is to develop specific scales per tasks to be explored. In this case, the explored task is the perceived clinical self-efficacy in mental health detection, treatment, and management at the level of primary care, particularly for the selected training modules. The developed self-efficacy questionnaire is thus comprised of questions aiming to understand GPs’ judgement of capabilities in detecting and diagnosing depression; psychosis; suicide/self-harm; alcohol use disorders; and drug use disorders; as well as treating and managing patients who present symptoms related to these disorders. An overall assessment that reflects self-efficacy will then be generated by averaging all the constructed domains of the scale.

A mental health practice questionnaire based on the *Mental Neurological and Substance Use Patient Visit Summary* developed by the WHO for the mhGAP Support and Supervision Guide will be administered. The purpose of administering this questionnaire is to collect the number of total cases (i.e., new, follow-up, or referred cases) before and after the training intervention, as well as patient socio-demographics.

Data will be collected at 4 times. At baseline (T-1, or before the training offered to the intervention group), GPs in both groups will be administered the 5 questionnaires (i.e., socio-demographic, knowledge, attitudes, self-efficacy and mental health practice). Post-intervention group training (T-2), both the intervention and control groups will be administered the same questionnaires, minus the socio-demographic questionnaire. The reason for the administration of the same questionnaires to the control group at T-2 is to account for contamination between groups during the intervention group training, and will serve as the pre-training measure for this group, also a way to maximize our sample. T-2 therefore is also known as the pre-test control group training measure. Post-test control group training (T-3), the control group will be administered the same knowledge, attitude, self-efficacy and mental health practice questionnaires. One year after the completion of the intervention group training (T-4), the groups will be administered the knowledge, attitude, self-efficacy and mental health practice questionnaires to assess whether the results of the training program were maintained over time.

#### Data management

JS, who is under the supervision of FC, NL, and MP, will be responsible for data collection, entry, coding, and management.

#### Statistical methods

All participants will be included in the analysis. This type of analysis is called intention-to-treat and is considered the best way to preserve the effects of randomization [53, 60]. Answers generated by questionnaires and surveys will be analyzed using SPSS Statistical Software (version 24).

T-tests on the difference in cluster means between the intervention and control groups [53] will be assessed for the questionnaires administered to the GPs. Two-tailed P values of less than 0.05 will be considered statistically significant. While the aforementioned t-tests take into account cluster level analysis, individual level analysis is discarded, which can underestimate the power of the analysis and generate misleading conclusions about the intervention [53, 61]. Adjustments can be made to the t-tests in order to account for individual level analysis. Campbell et al. (2000) [61] suggest that the t-test values (i.e., the differences between groups) should be divided by the square root of the design effect (i.e., 1 + (m-1) ICC). Two-tailed P values of less than 0.05 will be considered statistically significant. Individual level analysis will result in a higher significance level, compared with cluster level analysis [61].

### Phase 3: Exploring factors that influence implementation

#### Design

Multiple case study design will be used to explore how contextual factors within and across delegations (i.e., the cases) influence the successful implementation and expected outcomes of a mental health training based on the mhGAP-IG (version 1.0). According to Yin (2014) [62], case studies are most often used in order to answer ‘how’ questions, generally about situations that the researcher cannot control. Delegations are thus boundaries subject to a wider, uncontrollable context. They have been clearly established, and have specific particularities that we wish to uncover [63].

According to Yin (2014) [62], multiple case study design is based on a mix of qualitative and quantitative evidence. For this reason, multiple sources of data will be used to answer the research question, including focus groups with GPs, as well as quantitative data generated from the cluster RCT. These findings will be triangulated to develop what Yin (2014) [62] calls ‘converging lines of inquiry.’

#### Theoretical framework

An implementation model is necessary to guide the multiple case study design. There are a number of implementation models currently in use [64–69]. However, it is important to note that there is currently no consensus on constructs that make up implementation models and outcome measures [65, 69, 70]. This lack of agreement is due to the fact that implementing interventions is a multifaceted process that “involves attention to a wide array of multi-level variables related to the innovation itself, the local implementation context, and the behavioral strategies used to implement the innovation” [69].

While consensus on constructs and outcome measures to be included in implementation models has not been established, Champagne (2016) [71] regroups pre-existing implementation models to create a model for which complex and multi-faced factors and processes are taken into account. For this reason, Champagne (2016)’s [71] model will be used to develop focus group questions, as well as analyze or sort the collected data.

#### Data collection

Focus groups with the trained GPs working in delegations assigned to the intervention group will be conducted to explore how contextual factors influence the successful implementation of the mental health training based on the mhGAP-IG (version 1.0) and impact desired outcomes. Seven to 10 trained GPs from the intervention group will be interviewed at a time, a number that has been said to facilitate discussion by all participants [63]. Therefore, 2 focus groups will be conducted, with a total of 14 to 20 trained GPs. Focus groups will be conducted in French by JS and audio recorded. Data will be collected at T-2 (post-intervention group training).

#### Data analysis

Focus group audio recordings will be transcribed by JS and analyzed using thematic analysis [72]. This type of analysis focuses on developing common themes that are represented in the data. Important to note is that multiple case study design allows for the development of themes within cases and cross-cases [62]. More specifically, themes developed within delegations that receive the training will be reported, and they will be compared cross-delegations.

According to Padgett et al. (2008) [72], there are varied approaches to thematic analysis. The preferred method for this trial is to generate themes from the data that reflect initial interview questions, consistent with practices in evaluation research [72, 73]. In other words, the interview guide developed from Champagne (2016)’s [71] implementation model will serve as a thematic template for coding, and will be used to develop a code book before the coding process begins [72]. Coding will be done in QDA miner software (version 4.1.27).

To ensure rigor in the data analysis process, the code book will be devolved by JS, and validated by FC, NL and MP. Independent coding will be done in QDA minor software (version 4.1.27), using the developed code book. Coding from two independent reviewers will be merged, generating a percentage score for inter-rater reliability.

## Discussion

The purpose of this trial is to implement and evaluate a training based on the mhGAP-IG (version 1.0) offered to GPs in 2 Tunisian governorates (i.e., Tunis and Sousse), in order to uncover important information regarding implementation process and study design. Generated information will aid in country-wide implementation and evaluation. This training comes at an opportune time, given that Tunisia is currently undergoing a health services reform, one of its main objectives being to further develop proximity health services to address the mental health treatment gap in the country [41, 42]. In addition, given the political unrest and economic hardships currently experienced in Tunisia, mental health issues are of great national concern. While Tunisia has a mental health system, the uneven distribution of services and deficits in training for staff cause significant barriers to accessible care [41, 43].

This trial makes several practical contributions. First, its main focus is to train GPs in the detection, treatment and management of patients consulting for specific mental health problems in Tunis or Sousse, given their often limited capacity to address mental illness. Involvement of members of the Tunisian Ministry of Health in the implementation of this training program has prompted its inclusion under the national mandate of the Committee for Mental Health Promotion in Tunisia. A training under this Committee’s leadership has been dormant for years. In addition, this training aims to help further integrate mental health into primary care by training non-specialists in mental health. With GPs playing such an important role in the healthcare system, this training will help better utilize available resources in the country in order to target the mental health treatment gap.

This trial makes several contributions to the literature. To our knowledge, this is the first attempt to evaluate a mental health training program using a RCT design in Tunisia; implement a training based on the mhGAP-IG in Tunisia; and one of the first attempts to implement and evaluate a training based on the mhGAP-IG in a French-speaking nation. The trial will thus help build research capacity in Tunisia and more generally in LMICs, currently under-represented in the mental health literature [7, 34]. This trial also compliments the effectiveness results with implementation analysis, a current priority in global mental health [7, 26, 34]. Acknowledging factors that influence the successful implementation of a training program generates understanding on how context, especially within a health services reform such as the one currently underway in Tunisia, influences desired outcomes [36].

Lessons learned from this trial (i.e., successes and challenges regarding implementation of the training and acceptability of the trial design) can also be of use to other LMICs interested in implementing and evaluating a mental health training program based on the mhGAP-IG; designing a cluster RCT to evaluate the mhGAP-IG; or exploring contextual factors that can influence the success of a training intervention and expected results in a low-resource setting.

## Abbreviations

EPHPP: Effective public health practice project
GPs: General practitioners
HICs: High-Income countries
ICC: Intra-Cluster correlation
IG: Intervention guide
LMICs: Low- and middle-income countries
mhGAP: Mental health gap action programme
mhGAP-IG: Mental health gap action programme intervention guide
RCT: Randomized controlled trial
PAHO: Pan-American Health Organization
WHO: World health organization
